# 

**Published:** 1892-10

**Authors:** 


### Ohio State Dental Society—Annual Meeting.

The next annual meeting of the Ohio State Dental Society will be held in Columbus, on the first Tuesday in December, 1892.

Efforts are being made to have a large gathering of the profession of the State. The Executive Committee as well as the officers are actively at work to secure a good meeting. A number of subjects will come up for consideration that are of interest to the entire profession, and every man in the State who has any interest in the profession and a desire for its progress should be present, each prepared to do his share of the work that shall be before it.

The subject of Education will have an importance for this meeting that has never occurred before, and this especially in view of the fact that four new dental colleges have been organized in the State within the last year and a half.

The amended law has been in operation for the last few months, and will come up for its share of consideration.

The World's Columbian Dental Congress, of course, will come up for consideration, and probably some important questions to be decided in reference to it. There will manifestly be work enough to occupy a session of four full days, and we suggest that all the members and all the visitors arrange their business so as to remain and work from the beginning to the end of the meeting.

### CONTENTS.

### THE DENTAL REGISTER,

A MONTHLY MAGAZINE DEVOTED TO THE INTERESTS OF THE

**DENTAL PROFESSION.**

*Terms Reduced to $2.00 Per Year. Single Copies, 25 Cents.*

**EDITED BY PROF. J. TAFT, D.D.S.**

—AND—

**Published by SAM'L A. CROCKER & CO., Ohio Dental and Surgical Depot.**

The volume commences in January and closes in December.

The REGISTER being issued monthly is always fresh, therefore Indispensable to the practitioner who desires to keep posted up with the new inventions and improvements which are daily developed. Neither expense nor effort will be spared to give value and variety to its contents. Articles will be illustrated with well executed engravings whenever they will add to the interest or assist to explanation.

Its extensive circulation and frequency of issue make it one of the BEST DENTAL ADVERTISING MEDIUMS in the United States.

All subscriptions must begin with January or July.

Local or special notices, five lines or less, will be inserted for $3 each insertion; over five lines, 50 cts. per line.

All advertising bills payable in advance, or at furthest, after the issue of the first number containing the advertisement. All transient advertisements to be paid for in advance.

☞CONTRIBUTIONS SOLICITED.

Exclusively Editorial Communications should be addressed to the Editor.

The latest number of the REGISTER may be ordered with or without other goods at any time, and all letters should be addressed to

SAM'L A. CROCKER & CO., Ohio Dental and Surgical Depot, Cincinnati. O.

